# Accuracy evaluation of combining gastroscopy, multi-slice spiral CT, Her-2, and tumor markers in gastric cancer staging diagnosis

**DOI:** 10.1186/s12957-022-02616-z

**Published:** 2022-05-11

**Authors:** Songbo Zhao, Yangang Bi, Zhenfang Wang, Fantao Zhang, Yang Zhang, Yongyun Xu

**Affiliations:** 1 Department of Radiology, Shengli Oilfield Central Hospital, Dongying, 257034 China; 2 CT Room, Dongying People’s Hospital, 317 Dongchengnanyi Road, Dongying, 257091 China; 3 CT Room, Shengli Oilfield Central Hospital, Dongying, 257034 China

**Keywords:** Gastric cancer, MSCT, Gastroscopy, Immunohistochemical markers, Tumor markers, Combined diagnosis

## Abstract

**Background:**

To evaluate the diagnostic accuracy of single gastroscopy, multi-slice spiral CT, HER-2 or tumor markers, and their combination in the diagnosis of gastric cancer.

**Methods:**

A total of 98 patients with gastric cancer were selected as the research subjects. All patients underwent preoperative gastroscopy, MSCT, and the expression levels of HER-2, CEA, CA199, CA724, and CA242 were detected. A control group of 98 normal adults was selected to compare the risk factors for gastric cancer and to analyze the data.

**Results:**

There was statistical significance in the expression of the 5 markers in tumor size (*P* < 0.05), but no statistical significance in other clinical data (*P* > 0.05). The tumor marker CEA in gastric mucosal tissue of patients with gastric cancer had the highest positive detection rate for gastric cancer, and the difference was statistically significant (*P* < 0.05) compared with gastroscopy, MSCT and other markers. The combined diagnosis had higher sensitivity, specificity and accuracy compared with the single diagnosis of gastric cancer staging, and the difference was statistically significant (*P* < 0.05). Compared with normal adults, patients with gastric cancer had statistically significant differences in diet, body mass index, and family genetic history (*P* < 0.05), while there was no statistically significant difference in whether they had type A blood (*P* > 0.05).

**Conclusion:**

The combined diagnosis of gastroscopy, MSCT, immunohistochemical marker Her-2, and tumor markers CEA, CA199, CA724, and CA242 can more accurately determine the clinical staging and lesion invasion depth of patients with gastric cancer and can significantly improve the sensitivity of diagnosis.

## Introduction

Gastric cancer is one of the most common malignant tumors of digestive tract in the worldwide. In China, its morbidity and mortality are high and the annual mortality ranks first among all tumors, with the annual mortality as high as 0.02% [1]. Many methods are available for the treatment of gastric cancer, including surgery, radiotherapy, and chemotherapy. Most patients have unobvious symptoms in the early stage of the disease, which is easily ignored by patients and doctors. As a result, the patients were already in the middle or late stage when they were diagnosed through the typical symptoms later, and the best time for surgical treatment was missed. At this time, only radiotherapy and chemotherapy could be carried out, and the overall effect was not ideal [2]. Therefore, it is of great clinical significance to take active measures to accurately judge the specific stages of gastric cancer, formulate a reasonable treatment plan, and improve the prognosis for better treatment and prolong the survival time of patients [3]. And the accurate diagnosis of early preoperative staging in patients with gastric cancer has important clinical value.

In recent years, multi-slice spiral CT (MSCT) has been widely used in gastric cancer staging [4], with high accuracy in the invasion depth, invasion of organs around, and lymph node metastasis. Gastroscopy [5] is generally recognized as the best method for the diagnosis of gastric cancer, especially for the early staging. Gastroscopy shall present clear observations, and lesions of gastric mucosa can be directly observed, especially for those bulging, swelling, and ulcer lesions [6]. It is currently agreed that tumor markers in humans are expressed earlier than clinical symptoms. Therefore, tumor markers can be important indicators and play important roles in the diagnosis of gastric cancer, which can distinguish tumor tissues from those normal and determine the presence or absence of tumor [7, 8]. Most serum tumor markers have low sensitivity in detecting gastric cancer, affecting the accuracy of early diagnosis. Human epidermal growth factor receptor 2 (HER-2) is a proto-oncogene in human body, which has been found by some scholars to be related to the occurrence and progression of gastric cancer, and can also be used as an important marker for early diagnosis, treatment, and prognosis of this disease, with great significance [9, 10]. In this study, the combined detection of gastroscopy, MSCT, immunohistochemical marker HER-2, and tumor markers of carbohydrate antigen 199 (CA199), CA242, CA724, and carcino-embryonic antigen (CEA) was performed to observe and analyze the clinical effect of the diagnosis of gastric cancer staging, providing a basis for clinicians to select a reasonable treatment plan, with the report below.

## Methods

### General data

A total of 98 patients with gastric cancer diagnosed by postoperative pathology admitted to our hospital from October 2017 to May 2019 were selected as the subjects, including 63 males and 35 females, aged from 27 to 69 years old, with an average of 47.62 ± 17.16 years old. TNM staging: 29 in stage I, 37 in stage II, 21 in stage III, 11 in stage IV; lymph node metastasis: 58 had metastasis and 40 had no metastasis.

### Inclusion criteria

(1) Meeting the diagnostic criteria for gastric cancer in the *Guidelines for the Standardized Diagnosis and Treatment of Gastric Cancer*; (2) having no surgery or radiotherapy before the examination; (3) all being diagnosed with gastric cancer by biopsy pathology; (4) the patients and their families being informed and agreed to the study.

### Exclusion criteria

(1) Having diseases combined, such as endocrine system disease, immune system disease, blood system disease, and other diseases that may interfere with the detection of tumor markers; (2) patients having incomplete case data. The study was approved by the Ethics Committee of our hospital and informed consent was given by the patients and their families.

### Examination method

#### MSCT scanning

The GE Optima 660 64 Slice CT scanner was adopted. The patients fasted for 8 h before the examination, and received an intramuscular injection of 20 mg anisodamine (Fujian Sanai Pharmaceutical Co., Ltd., Guoyao Zhunzi H35020158) 10−20 min before the examination, and 800−1000 mL of drinking water to fill the gastric cavity. The patients were scanned in a supine, side or prone position, scanning from the top of the right diaphragm to the diaphragmatic crest, including the whole abdomen and pelvic cavity. Eighty milliliters of iopromide (Bayer Schering Pharma AG, Germany, Guoyao Zhunzi: J20130157) was injected into the patient’s elbow vein through a high-pressure syringe at a flow rate of 3.5 mL/s. After 30 s of injection of contrast agent, enhanced arterial scanning was performed, scanning from the esophagus, abdomen to whole stomach. After 60 s of injection of contrast agent, intravenous scanning was performed to observe the location of the lesion, its organs around and the distal metastasis, etc. The scanning parameters were as follows: voltage 120 kV, current 250 mA, layer thickness 5 mm, spacing 5 mm, and pitch 1.625 mm.

#### Gastroscopy

Olympus Gastrointestinal Videoscope GIF-HQ290 was adopted. The patients fasted for 8 hours before the examination. In order to avoid reflex vomiting during the examination, 5~10 min before the examination, the patients took dyclonine hydrochloride mucilage orally (Yangtze River Pharmaceutical Group, Guoyao Zhunzi H20041523) to anesthetize the throat, took the mouth gag, and kept the left side lying. The gastrointestinal endoscope was then used to enter the mouth, pass through the trachea and esophagus, and then reach the inside of the stomach to observe and diagnose the internal tissues. Lesions with hard texture, solitary erosion or crater shape observed under gastroscopy were collected for biopsy. In case the lesion had a diameter of ≤ 1.0 cm, then all samples were taken; or the lesion > 1.0 cm, then the selected sampling shall be completed.

#### Immunohistochemical markers

Gastric mucosa specimens of all patients were collected through endoscopy, fixed with 3.7% neutral formaldehyde solution, routinely dehydrated, embedded in paraffin, cut into slices of 4 μm, baked and dewaxed. Immunohistochemistry was used to detect Her-2 expression in gastric cancer tissues. The kit was purchased from Shanghai Huzhen Biological Technology Co., Ltd. The MaxVision two-step method was used for dyeing. The color development reagent DAB solution was added, lasted for 1 to 2 min, dyed with hematoxylin, dehydrated, and sealed with neutral transparent gum. Phosphate-buffered saline (PBS) was used instead of the primary antibody, as a negative control group. Cell membrane showing brown-yellow granular precipitation was taken as the judgement criteria. For no or < 10% cells having staining, expressed as 0; for ≥ 10% cells having slight staining, expressed as +; for ≥ 10% cells having weak staining, expressed as ++; for ≥ 10% cells having medium to intensive staining, expressed as +++. (0) and (+) indicated low expression, that was, Her-2 was negative; (++) and (+++) indicated overexpression, that was, Her-2 was positive.

#### Tumor marker detection

Before surgery, 6 mL of the venous blood in the morning under the fasting state of the patient was collected, centrifuged at 1500 r/min for 15 min, and the supernatant was taken. ELISA was adopted to detect tumor markers, including carcinoembryonic antigen (CEA), carbohydrate antigen 242 (CA242), carbohydrate antigen 724 (CA724), and carbohydrate antigen 199 (CA199). All kits were purchased from Shanghai Huzhen Biotechnology Co., Ltd. The positive standards for CEA, CA242, CA724, and CA199 were > 5 ng/mL, > 25 U/mL, > 6.9 U/mL, and > 35 U/mL, respectively.

#### ROC curve of factors causing gastric cancer

Ninety-eight patients suffering from gastric cancer and 98 normal adults were statistically analyzed for their diet, BMI, family genetic history, and type A blood.

#### Image processing

The examination results of all patients were read by two attending radiologists; in case of any dissension, it shall be adopted after consultation.

### Indicators

All patients with gastric cancer were classified into, according to the TNM staging [11], I, II, III, and IV; and the materials regarding the gender, age, tumor size, lesion location, and lymph node metastasis was collected. The sensitivity, specificity, and accuracy of single detection by gastroscopy, MSCT, HER-2, or tumor marker, and their combination of all patients were observed for comparation. According to AJCC TNM Staging System for Gastric Cancer 2016, T1: tumor invades the muscularis mucosae or submucosa; tumor invades the muscularis propria; T3: tumor penetrates the subserosal connective tissue without invasion of the visceral peritoneum or adjacent structures; tumor invades the serosa or adjacent structures.

### Criteria for the positive result of comprehensive detection

Gastroscope, MSCT, Her-2, CEA, CA242, CA724, and CA199 were combined for the detection of gastric cancer. If two of the above results were positive, the comprehensive result was positive; otherwise, it was negative.

### Statistical analysis

SPSS 23.0 software was used for statistical analysis of all data. Measurement data was expressed as mean ± standard deviation (‾x ± s). Counting data was expressed in percentage (%). *χ*^2^ test was for comparison between the 2 groups, and pathological results were taken as the gold standard. Differences in the accuracy of single MSCT, gastroscopy, immunohistochemical marker HER-2, tumor marker, and combined examination in the evaluation of clinical staging of gastric cancer were analyzed, and *P* < 0.05 was considered statistically significant.

## Results

### Comparison of the correlation between immunohistochemical markers and tumor markers and clinicopathological indicators of gastric cancer

Among 98 patients with gastric cancer, 25 had positive Her-2 expression, accounting for 25.5%; 35 had positive CEA expression, accounting for 35.71%; 27 had positive CA724 expression, accounting for 27.55%; 15 had positive CA242 expression, accounting for 15.31%; and 22 had positive CA199 expression, accounting for 22.45%. For the positive rates of these 5 markers, the difference in tumor size was statistically significant (*P* < 0.05), and the differences in gender, age, lesion location, and lymph node metastasis were not statistically significant (*P* > 0.05) (Table 1).

**Table 1:** Comparison of the correlation between immunohistochemical markers and tumor markers and clinicopathological indicators of gastric cancer (*n*)

	Indicators	Her-2	CEA	CA724	CA242	CA199	*χ*^2^	*P*
Gender	M	16	22	17	10	14	0.075	> 0.05
	F	9	13	10	5	8		
Age (years)	≤ 60	11	14	12	8	9	0.837	> 0.05
	> 60	14	21	15	7	13		
Tumor size (cm)	≤ 5	9	12	16	9	8	6.63	> 0.05
	> 5	16	23	11	6	14		
Lesion location	Gastric fundus	4	5	5	1	2	4.68	> 0.05
	Gastric body	5	7	3	3	4		
	Gastric antrum	8	13	12	7	10		
	Gastric Fundus, and body	3	4	3	1	2		
	Gastric body and antrum	3	3	2	2	3		
	Total carcinoma of stomach	2	3	2	1	1		
Lymph node metastasis	Yes	14	18	16	8	14	0.97	> 0.05
	No	11	17	11	7	8		

### Staging results of gastric cancer diagnosed by gastroscopy, as shown in Fig. 1

**Fig. 1:**
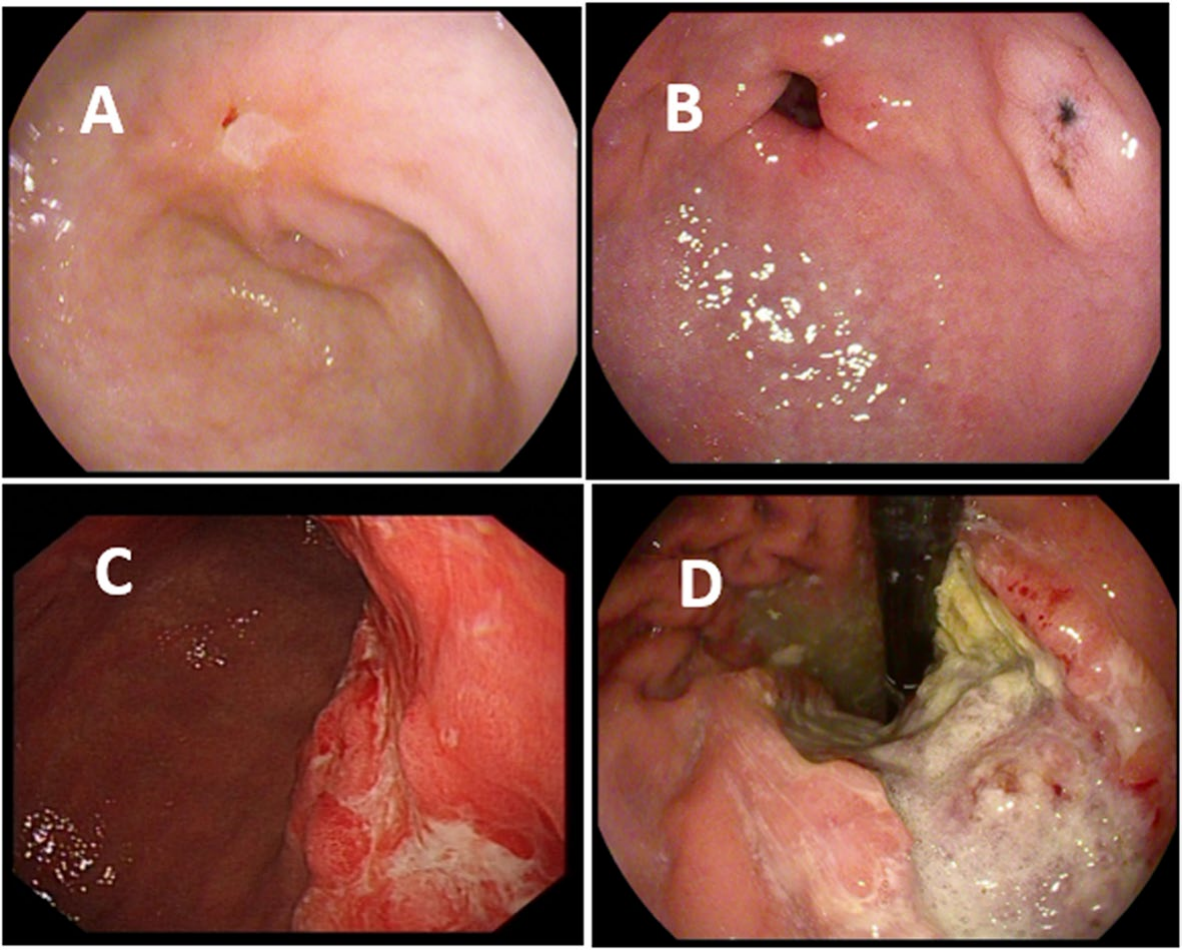
Results of gastroscopy. **A** The gastric cancer in stage I, manifesting as gastric antrum mucosal congestion and edema, irregular ulcers, surrounding mucosa protrusion, poor elasticity in biopsy, and easy bleeding. **B** The gastric cancer in stage II, manifesting as irregular ulcer on the anterior wall of gastric antrum, surrounding mucosa protrusion, poor elasticity in biopsy, and easy bleeding. **C** The gastric cancer in stage III, manifesting as huge ulcerative lesion in gastric antrum, slight protrusion of surrounding mucosa, surface hyperemia and edema, and tough quality in biopsy. **D** The gastric cancer in stage IV, manifesting as huge ulcerative lesions in the gastric body, surface congestion, edema, erosion, covered with dirty of purulent surface, and fresh bleeding, fragile quality in biopsy, and poor elasticity

Figure 1A shows the gastric cancer in stage I, manifesting as gastric antrum mucosal congestion and edema, irregular ulcers, surrounding mucosa protrusion, poor elasticity in biopsy and easy bleeding. Figure 1B shows the gastric cancer in stage II, manifesting as irregular ulcer on the anterior wall of gastric antrum, surrounding mucosa protrusion, poor elasticity in biopsy, and easy bleeding. Figure 1C shows the gastric cancer in stage III, manifesting as huge ulcerative lesion in gastric antrum, slight protrusion of surrounding mucosa, surface hyperemia and edema, and tough quality in biopsy. Figure 1D shows the gastric cancer in stage IV, manifesting as huge ulcerative lesions in the gastric body, surface congestion, edema, erosion, covered with dirty of purulent surface, and fresh bleeding, fragile quality in biopsy, and poor elasticity.

### Gastric cancer staging from MSCT diagnosis, as shown in Fig. 2

**Fig. 2:**
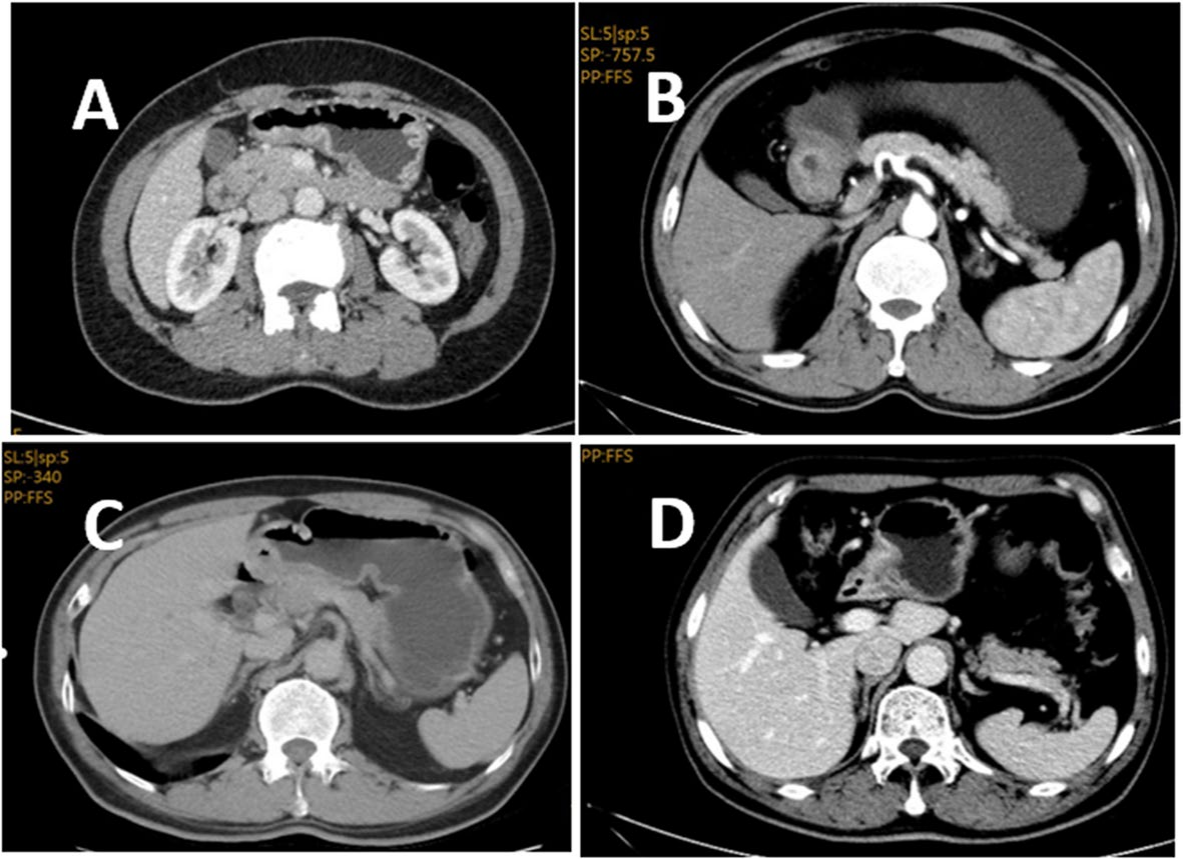
MSCT results. **A** Stage T1, manifesting as focal enhancement and thickening of the gastric wall, as well as an intact hypodensity zone in the submucosa. **B** Stage T2, manifesting as localized gastric wall thickening with smooth outer margin of gastric wall, and flocculent shadow in fat layer. **C** Stage T3, manifesting as irregular serosal surface at the outer boundary of the thickened gastric wall, blurred fat layer around the lesion, and nodules. **D** Stage T4, manifesting as rough gastric serosal mucosal surface, blurred space with fatty layer, and invasion of adjacent organs

Figure 2A shows stage T1, manifesting as focal enhancement and thickening of the gastric wall, as well as an intact hypodensity zone in the submucosa. Figure 2B shows stage T2, manifesting as localized gastric wall thickening with smooth outer margin of gastric wall, and flocculent shadow in fat layer. Figure 2C shows stage T3, manifesting as irregular serosal surface at the outer boundary of the thickened gastric wall, blurred fat layer around the lesion, and nodules. Figure 2D shows stage T4, manifesting as rough gastric serosal mucosal surface, blurred space with fatty layer, and invasion of adjacent organs.

### The results of pathological examination, as shown in Fig. 3

**Fig. 3:**
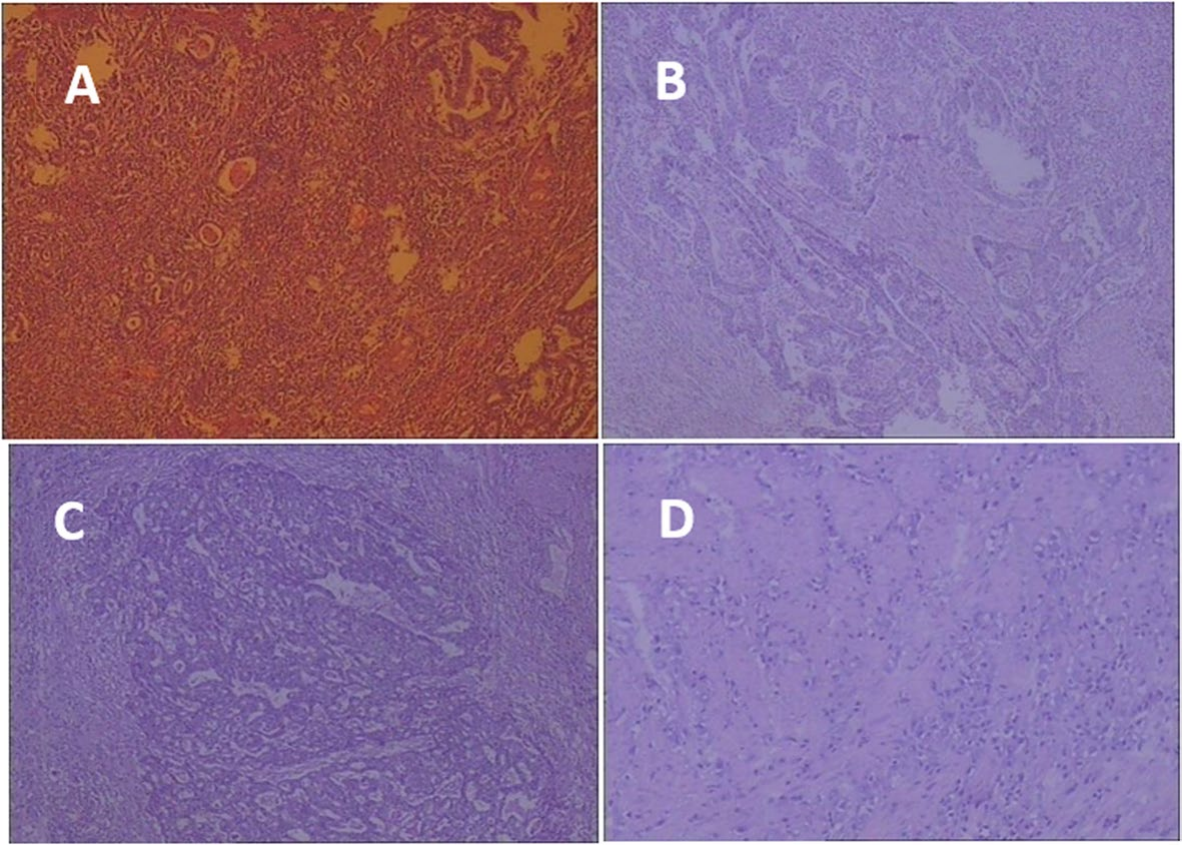
Results of pathological examination. **A** Stage T1, poorly differentiated adenocarcinoma of gastric ulcer-type, with signet ring cell carcinoma differentiated in small foci and invading submucosa. **B** Stage T2, moderately poorly differentiated adenocarcinoma of ulcerated protrusion-type, with tumor size of 5 × 4 × 2 cm, invasion depth of deep muscle, total lymph node metastasis of 31, nerve invasion. **C** Stage T3, moderately poorly differentiated adenocarcinoma of ulcerated protrusion-type in gastric antrum, with tumor size of 3 × 2.5 cm, partial differentiation of hepatoid adenocarcinoma being considered, the invasion of tumor infiltration in subserosal connective tissue, and 24 lymph node metastases. **D** Stage T4, ulcerative poorly differentiated adenocarcinoma, some mucinous cell carcinoma, infiltrating the serosal membrane and invading the nerve

Figure 3A shows stage T1, poorly differentiated adenocarcinoma of gastric ulcer-type, with signet ring cell carcinoma differentiated in small foci and invading submucosa. Figure 3B shows stage T2, moderately poorly differentiated adenocarcinoma of ulcerated protrusion-type, with tumor size of 5 × 4 × 2 cm, invasion depth of deep muscle, total lymph node metastasis of 31, nerve invasion. Figure 3C shows stage T3, moderately poorly differentiated adenocarcinoma of ulcerated protrusion- type in gastric antrum, with tumor size of 3 × 2.5 cm, partial differentiation of hepatoid adenocarcinoma being considered, the invasion of tumor infiltration in subserosal connective tissue, and 24 lymph node metastases. Figure 3D shows stage T4, ulcerative poorly differentiated adenocarcinoma, some mucinous cell carcinoma, infiltrating the serosal membrane and invading the nerve.

### Comparison of single and combined detection for diagnosis of gastric cancer clinical staging

The tumor marker CEA had the highest positive rate for each stage of gastric cancer, and was significantly higher than that of other markers, gastroscopy and MSCT (*P* < 0.05). MSCT, Her-2, CA724, and CA199 expression was significantly higher than those of CA242 and gastroscopy (*P* < 0.05). The combined detection in the diagnosis of gastric cancer staging had significantly higher sensitivity, specificity, and accuracy (*P* < 0.05) than those of the individual detections, as shown in Table 2 and gastroscope in Table 3.

**Table 2:** Comparison of single and combined detection for diagnosis of gastric cancer clinical staging (*n*)

Clinical staging	Cases	Her-2	CEA	CA724	CA242	CA199	Gastroscope	MSCT	Combined detection
I	29	4	7	4	2	4	2	5	23
II	37	8	10	8	5	7	4	9	32
III	21	7	10	8	5	6	5	9	19
IV	11	6	8	7	3	5	3	7	11
Total	98	25	35	27	15	22	14	30	85

**Table 3:** Comparison of sensitivity, specificity, and accuracy of single and combined detection for diagnosis of gastric cancer clinical staging (%)

	Clinical staging	Her-2	CEA	CA724	CA242	CA199	Gastroscope	MSCT	Combined detection
Sensitivity	I	13.79 (4/29)	24.14 (7/29)	13.79 (4/29)	6.90 (2/29)	13.79 (4/29)	6.90 (2/29)	17.24 (5/29)	79.31 (23/29)
	II	21.62 (8/37)	27.02 (10/37)	21.62 (8/37)	13.51 (5/37)	18.92 (7/37)	10.81 (4/37)	24.32 (9/37)	86.49 (32/37)
	III	33.33 (7/21)	47.62 (10/21)	38.10 (8/21)	23.81 (5/21)	28.57 (6/21)	23.81 (5/21)	42.86 (9/21)	90.48 (19/21)
	IV	54.55 (6/11)	72.73 (8/11)	63.64 (7/11)	27.27 (3/11)	45.45 (5/11)	27.27 (3/11)	63.64 (7/11)	100
Specificity	I	(11/11)							
	II	72.46 (50/69)	82.61 (57/69)	69.57 (48/69)	60.87 (42/69)	60.87 (42/69)	63.77 (44/69)	73.91 (51/69)	97.10 (67/69)
	III	60.66 (37/61)	62.30 (38/61)	55.74 (34/61)	52.46 (32/61)	62.30 (38/61)	57.38 (35/61)	62.30 (38/61)	90.16 (55/61)
	IV	68.83 (53/77)	70.12 (54/77)	75.32 (58/77)	71.43 (55/77)	72.73 (56/77)	67.53 (52/77)	71.43 (55/77)	96.10 (74/77)
Accuracy	I	93.10 (81/87)	94.25 (82/87)	95.40 (83/87)	94.25 (82/87)	94.25 (82/87)	90.80 (79/87)	94.25 (82/87)	97.70 (85/87)
	II	55.10 (54/98)	65.31 (64/98)	53.06 (52/98)	44.90 (44/98)	46.94 (46/98)	46.94 (46/98)	57.14 (56/98)	91.84 (90/98)
	III	45.92 (45/98)	48.98 (48/98)	42.86 (42/98)	37.76 (37/98)	45.92 (45/98)	39.80 (39/98)	47.96 (47/98)	88.78 (87/98)
	IV	61.22 (60/98)	65.31 (62/98)	67.35 (66/98)	61.22 (60/98)	63.27 (62/98)	58.16 (57/98)	65.31 (64/98)	94.90 (93/98)
Overall accuracy		62.76	88.78 (87/98)	91.84 (90/98)	91.84 (90/98)	86.73 (85/98)	88.78 (87/98)	83.67 (82/98)	90.82 (89/98)

**Fig. 4:**
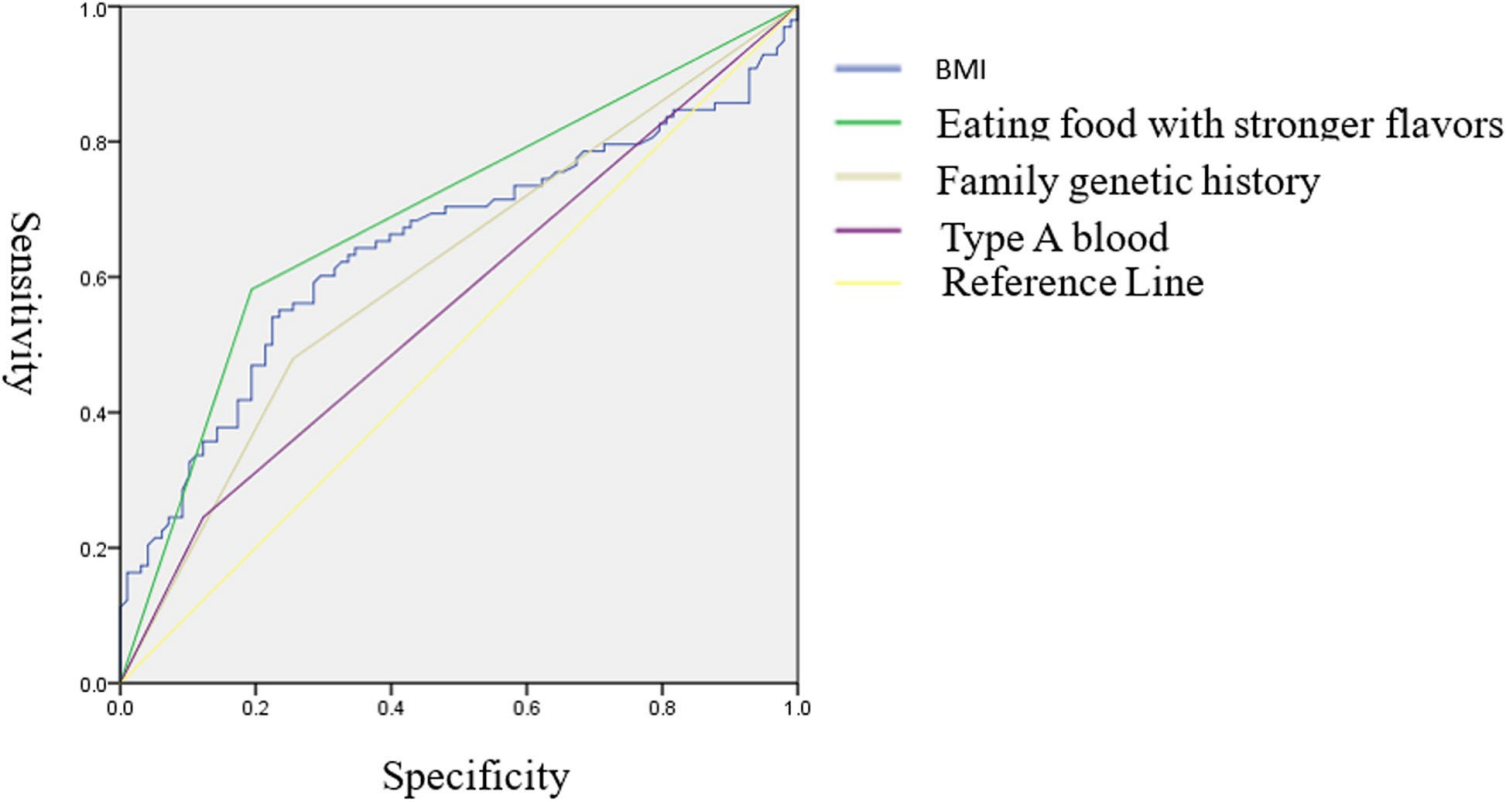
Analysis of factors causing gastric cancer ROC curve

Analysis of factors causing gastric cancer ROC curve results showed that eating food with stronger flavors, BMI, and family genetic history were important factors affecting gastric cancer, and the most influential was eating food with stronger flavors (Fig. 4, Table 4).

**Table 4:** Area under the curve

Variable	Interval	Standard deviation	Sig	95% confidence interval	
				Lower-bound	Upper-bound
BMI	0.647	0.040	0.000	0.569	0.726
Eating food with stronger flavors	0.694	0.038	0.000	0.619	0.769
Family genetic history	0.612	0.040	0.007	0.533	0.691
Type A blood	0.561	0.041	0.139	0.481	0.642

## Discussion

Nowadays, gastric cancer has been one of the important diseases that pose a serious threat to people’s physical and mental health and quality of life, with high incidence and mortality [12]. In clinic practices, in most cases imaging and pathology are used to diagnose patients suffering from tumor. In recent years, as tumor marker detection has been widely used, more serum tumor markers detection is common for tumor patients, and further prognostic and diagnostic effect evaluation are conducted [13]. A high-quality tumor marker should not only have strong tumor specificity, but also be able to quantitatively evaluate the tumor response load, and then detect the tiny lesions. In tumor detection, many immunohistochemical and tumor markers are available to diagnose gastric cancer, but with poor sensitivity and specificity, and the disease cannot be accurately diagnosed in the early stage [14]. Non-specific serum tumor markers can also have a certain value in the evaluation and diagnosis of gastric cancer staging [15]. As medical experts have conducted in-depth research on markers, more and more new tumor markers have been discovered for tumors detection, and the importance of immunohistochemical markers in the pathological diagnosis of gastric cancer has gradually been accepted by people [7, 9]. Currently, specific immunohistochemical indicators are widely used in clinical practices, which can improve the early diagnosis rate for patients with gastric cancer [16]. In this study, for 98 patients with primary gastric cancer, tumor markers and immunohistochemical indicators were detected and analyzed, and the results showed that the tumor marker CEA had the highest positive detection rate, and significantly higher than that of other markers (*P* < 0.05).

It is currently agreed that in clinic practices, the key to improving the treatment effect and prognosis for patients with gastric cancer is early screening and diagnosis. Studies have found [17] that tumor markers have played important roles in the occurrence and progression of tumors, and can be used for diagnosis, with common ones including CEA, CA242, CA724, CA199, etc. Among them, CEA protein is rich in polysaccharides and has human embryonic antigen specificity. Relevant research data have shown that CEA has been widely used in the diagnosis of various tumor diseases, with its level increased significantly in gastrointestinal tumors [18]. In this study, CEA had a detection sensitivity of 35.71% and sensitivity 78.57%, and patients in stage III, IV, and lymph node metastasis had a higher CEA positive rate, showing that CEA expression was possibly related to the staging and lymph node metastasis. CEA can make the cell arrangement disorder, change the location of cancer cells, and then may cause infiltration and metastasis [19]. CA724 is a mucin with high molecular weight and is highly expressed in some tissues, such as human embryo tissues and some malignant tumors, but is weakly expressed in normal differentiated tissues [20]. CA199 had low specificity in the diagnosis of patients with gastric cancer. Reports have shown that CA199 had a certain correlation with tumor size and lymph node metastasis, and could be used as a reliable indicator for the diagnosis and prognosis of patients with gastric cancer, but the sensitivity was not as good as CEA, this result was consistent with that of this study [21]. CA242 is an acidified mucin, mainly found in embryonic tissues, having the expression level not high in the serum of normal people and patients with benign diseases, with the content increased significantly in the lesions of malignant gastrointestinal tumors, so it can be one of the indicators for malignant tumor diagnosis [22]. From this study, the diagnostic sensitivity of CA242, CA724, and CA199 to gastric cancer were 15.31%, 27.55%, and 22.45%, respectively, the specificity were 71.77%, 75.85%, and 74.15%, respectively. The sensitivity and specificity of the four tumor markers in the diagnosis of gastric cancer were higher in stages III–IV and lower in stages I−II, which may be that the change is not significant in the content of the tumor markers in the early stage of cancer, resulting in a lower positive detection rate. With the change of the disease condition, there were significant changes of the marker content in the patient’s serum, and abnormality can be easily detected, so the detection rate is higher. There were certain differences in the positive rates of CA242, CA724, and CA199 in patients with different clinical staging and lymph node metastasis, so it can be inferred that these indicators also had a certain value in the diagnosis of gastric cancer.

As a cell proto-oncogene, HER-2 gene is a member of the epidermal growth factor receptor (HER) family, mainly located in the cell membrane and expressed in a small amount in the cytoplasm, playing a certain role in the normal division and growth of cells [23]. HER-2 has weak expression in normal adult tissues, but under pathological conditions, its overexpression can not only inhibit tumor cell apoptosis, but also promote tumor cardiovascular regeneration and increase tumor cell invasion [24]. Studies have shown [25] that HER-2 gene amplification, RNA and protein overexpression are common in gastric cancer, but these cannot be detected in non-cancer tissues, indicating that HER-2 plays an important role in the process of angiogenesis, invasion, and metastasis, and can affect the proliferation, differentiation, metastasis and adhesion of tumor cells. In this study, Her-2 showed an expression sensitivity of 25.51%, and the positive rate of Her-2 expression in patients with different clinical stages and lymph node metastasis is statistically significant (*p* < 0.05). Therefore, it can be inferred that the abnormal elevation of the immunohistochemical marker HER-2 may indicate the occurrence of early gastric cancer or precancerous lesions, which can assist in the early diagnosis of gastric cancer, and contribute to the accurate evaluation of clinical staging, judgment of lymph node metastasis and prognosis. In this study, the number of positive cases detected by Her-2, CEA, CA242, CA724, and CA199 was compared, and their relationship with clinicopathological characteristics was compared and analyzed. The difference was not statistically significant (*P* > 0.05), indicating that the positive rate of Her-2, CEA, CA242, CA724, and CA199 in detecting gastric cancer is not correlated with clinicopathological features.

Studies have found [26] that gastroscopy can make the preoperative histological types and differentiation degree clear, but cannot evaluate the clinical staging of gastric cancer, especially for early gastric cancer with small lesions. For some mucinous adenocarcinomas and signet-ring cell carcinomas mainly characterized by submucosal infiltration, the mucosal layer in the lesion area is usually relatively intact. If the cancer cells infiltrate along the submucosal layer, gastroscopy may not detect them, leading to misdiagnosis or missed diagnosis [27, 28]. Many methods for preoperative diagnosis of gastric cancer are currently available, including MSCT, tumor staging, efficacy evaluation, etc. [29]. In recent years, MSCT has improved both in time and space resolution along the continuous development of medical imaging, and has advantages in evaluating the depth of tumor invasion of the stomach wall, lymph node metastasis, and adjacent organ infiltration in patients with gastric cancer [30]. Studies have also found [31] MSCT may cause a higher missed diagnosis and misdiagnosis due to its lower sensitivity to smaller lesions or metastatic lesions below 5 mm, and it is easy to diagnose lymph node inflammation as metastasis, leading to a certain deviation between the results and the pathological diagnosis.

## Conclusion

In conclusion, combining the gastroscopy, MSCT, immunohistochemical marker HER-2, serum tumor marker CEA, CA242, CA724, and CA199 can improve the sensitivity of early diagnosis for gastric cancer, showing a sensitivity of 86.73%, and significantly better than that of each single detection (*P* < 0.05). This indicates that the combined detection plays a positive role in the early diagnosis of patients suffering from gastric cancer, which can significantly make the diagnosis accuracy improved, assist clinicians to formulate reasonable surgical treatment, reduce unnecessary abdominal laparotomy, and greatly improve the quality of treatment.

## Data Availability

The datasets used and/or analyzed during the current study are available from the corresponding author on reasonable request.
